# Exploratory Study of the Phase IV Immunization Schedule of Quadrivalent Influenza Split-Virion Vaccine in Children Aged 3–8 Years

**DOI:** 10.3390/vaccines12030321

**Published:** 2024-03-18

**Authors:** Xiaoyu Li, Zengqiang Kou, Ti Liu, Wenjue An, Wenqi An, Wei Zhang, Ke Zhang, Jie Dong, Jiangxuan Yu, Yaqi Li, Chenyan Zhao

**Affiliations:** 1 Division of HIV/AIDS and Sex-Transmitted Virus Vaccines, National Institutes for Food and Drug Control, Beijing 102629, China; lixiaoyu@nifdc.org.cn; 2 Institute for the Prevention and Control of Infectious Diseases, Shandong Center for Disease Control and Prevention, Jinan 250013, China; jack-cou@163.com (Z.K.); liuti1204@126.com (T.L.); 3 Hualan Biological Bacterin Inc., Xinxiang 453003, China; awj4243@hualanbacterin.com (W.A.); awq2139@hualan.com (W.A.); zw0572@hualanbacterin.com (W.Z.); zk0386@hualanbacterin.com (K.Z.); dj0587@hualanbacterin.com (J.D.); yjx6714@hualanbacterin.com (J.Y.); lyq6392@hualanbacterin.com (Y.L.)

**Keywords:** quadrivalent influenza split-virion vaccine, immunogenicity, non-immunization history, 3–8 years of age, immunization schedule

## Abstract

This study explored the optimum immunization schedule for the quadrivalent influenza split-virion vaccine containing influenza A strains (H1N1 and H3N2) and B lineage strains (Yamagata and Victoria) in children aged 3–8 years. The 652 participants enrolled were divided into two groups based on a history of influenza immunization (IH group) or no history of influenza immunization (NH group). The groups were administered a two-dose immunization schedule on days 0 and 30. In the NH group, on day 30 after the first dose, the positive rates of influenza hemagglutination-inhibition antibodies of strains H1N1, H3N2, BV, and BY were 85.85%, 71.70%, 65.27% and 60.45%, respectively. The positive rates of BV and BY failed to meet the absolute criteria for evaluating the immunogenicity of influenza vaccine in the population aged 3–60 years (for each strain antibody). On day 30 after the second dose, HI antibodies to strains H1N1, H3N2, BV, and BY met the immunogenicity acceptable criteria. In the IH group, on day 30 after the first dose, HI antibodies to strains H1N1, H3N2, BV, and BY met the acceptable criteria for immunogenicity. The incidence rates of adverse reactions (vaccine-related adverse events) from the first dose up until 30 days after the second dose were 20.80% in the IH group and 19.50% in the NH group. Only two Grade 3 adverse reactions (fever: temperature ≥ 39.5 °C, swelling: area ≥ 50% of the injection site area) occurred in the IH group, and no Grade 3 adverse reactions occurred in the NH group. No serious adverse reactions occurred in either group. We conclude that for the NH group, two doses of quadrivalent influenza vaccine should be administered, and for the IH group, a one-dose regimen is acceptable.

## 1. Introduction

Influenza (commonly known as the “flu”) is a zoonotic acute respiratory infectious disease that usually presents a seasonal epidemic pattern in China [1]. During an influenza epidemic, school-age children show high levels of morbidity [2], directly or indirectly causing significant economic losses. Administering influenza vaccines is recognized as the most effective means to prevent influenza, respiratory-related diseases, and serious complications such as pneumonia, myocarditis, and encephalitis [3].

Until June 2023, the vaccination schedule for approved inactivated influenza vaccines in China for individuals aged 3 years or older was one dose before or during each influenza epidemic season. However, the guidelines for influenza vaccination in China and other countries suggest that children 3–8 years of age should be administered vaccines on different immunization schedules according to their vaccination history [4,5]. The children aged 3–8 years who have previously received ≥2 total doses of trivalent or quadrivalent influenza vaccine ≥4 weeks apart require only one dose. Those who have not previously received ≥2 doses of trivalent or quadrivalent influenza vaccine ≥4 weeks apart require two doses.

The aim of our study was to explore the optimum immunization schedule for children aged 3–8 years based on up-to-date research. Hualan Biological Bacterin Inc. (hereafter referred to as “Hualan Bio”) refers to the influenza vaccination advice presented in “Prevention and Control of Seasonal Influenza with Vaccines: Recommendations of the Advisory Committee on Immunization Practices—United States, 2020–21 Influenza Season [5]” issued by the Advisory Committee on Immune Practice in the United States (ACIP) and “Technical Guidelines for Influenza Vaccination in China (2020–2021) [4]” issued by the China CDC. In our study, we recruited children aged 3–8 years and divided them into groups based on their influenza immunization history. This research involved a self-control and intergroup-control design and carried out an immunization program in children aged 3–8 years using the quadrivalent influenza vaccine, which contained the hemagglutinin of influenza A strains (H1N1 and H3N2) and B lineage strains (Yamagata and Victoria), was administered intramuscularly into the lateral deltoid muscle of the upper arm. Children in the immunization history (IH) group had received two or more doses of influenza vaccine cumulatively in the past, and children in the no immunization history (NH) group had not received the vaccine or had received only one dose of influenza vaccine in the past. This study aimed to explore the optimum immunization schedule for the quadrivalent influenza vaccine in children aged 3–8 years.

## 2. Materials and Methods

### 2.1. Study Design

A randomized and open trial was conducted to explore a science-based immunization schedule for the administration of the quadrivalent influenza split-virion vaccine manufactured by Hualan Biological Bacterin Inc. in children aged 3–8 years in Dong’e County, Shandong, China, from 15 September 2021 to 22 August 2022. Shandong Center for Disease Control and Prevention was responsible for implementing this research. The National Institutes for Food and Drug Control tested the serum antibodies of the trial. The data manager and statistical analyst were from Zhengzhou Maidike Pharmaceutical Technology Co., Ltd. Before initiation of the research, the research plan, an informed consent form (ICF), and other information offered to recipients were approved by the Shandong CDC Medical Ethics Committee (MEC) (Approval number: Lunyanpi NO 2021-62). Clinical trial data are published on the Drug Clinical Trial Registration and Information Disclosure Platform (www.chinadrugtrials.org.cn, accessed on 18 March 2024) (No. CTR20212369).

### 2.2. Study Population

Volunteers were screened based on the inclusion and exclusion criteria approved by the Medical Ethics Committee. The inclusion criteria were as follows. (1) Healthy participants aged 3–8 years old with legal identification who permanently reside in Dong’e County. (2) Their guardians voluntarily agreed to participate in this study and signed an informed consent form (ICF). They can also comply with the requirements of the clinical trial plan. (3) Recipients are deemed healthy through medical history inquiries, physical examination, and clinical diagnosis and are eligible for immunization with the quadrivalent influenza vaccine. The exclusion criteria were as follows. (1) Subjects have been administered any influenza vaccine within the 6 months before enrollment (including any registered or research vaccine) or plan to receive such a vaccine during the research period. (2) Are allergic to any vaccine component or report an allergic response to any vaccine. (3) Have received immunoglobulin and/or any other blood products within the 3 months before enrollment or plan to receive any such products during the research period (before post-immunization blood sample collection). (4) Have received immune-boosting or immunosuppressive medication during the previous 3 months (either continuous oral administration or infusion for more than 14 days). (5) Have been administered a live attenuated vaccine within the previous 14 days or any other vaccine within the previous 7 days. (6) Have a history of Guillain–Barre syndrome (GBS) when inoculated against influenza vaccine. (7) Have a preexisting acute disease, severe chronic disease, experiencing an acute attack of a chronic disease, or currently infected with influenza. (8) Epilepsy that is not controlled medically. (9) A diagnosis of congenital or acquired immunodeficiency, HIV infection, lymphoma, leukemia, or other autoimmune disease. (10) A history of abnormal coagulation function (e.g., deficiency of coagulation factor, coagulation disease). (11) Primary and secondary immunocompromised patients (a history of excision of the thyroid, pancreas, liver, or spleen). (12) A history of a serious allergic reaction to vaccination. (13) Participating in or planning to participate in another clinical trial. (14) An axillary temperature of ≥37.5 °C before administering the vaccine. (15) The researchers deem that some other factor compromises eligibility for this clinical trial.

### 2.3. Study Vaccine

The vaccine used in the research was the quadrivalent influenza split-virion vaccine commercially produced by Hualan Bio (batch number 202106A013). Each formulation delivered 15 µg of hemagglutinin of each component strain per dose. The constituent strains as recommended by WHO for 2021–2022 were A/Victoria/2570/2019 (H1N1)pdm09-like, A/Cambodia/e0826360/2020 (H3N2)-like, B/Washington/02/2019 (B/Victoria lineage)-like and B/Phuket/3073/2013 (B/Yamagata lineage)-like. Vaccines were stored and transported at 2–8 °C, avoiding freezing. 

### 2.4. Sample Size

An open design, using the PASS 15.0 confidence interval of one proportion (CI) method, was used to estimate the sample size based on the antibody seroconversion rate and the antibody positive rate. Each group was estimated to require 277 cases. Considering the possible dropouts, the sample size of each group was expanded to 326 cases, and the total sample size was 652 cases. 

### 2.5. Study Population, Grouping, and Vaccination

Influenza vaccination history was checked in the official vaccination records of each participant. A blind method was not used. After screening, subjects were assigned into two groups based on their vaccination history, i.e., those with a history of influenza immunization, the immunization history (IH) group, and those with no history of influenza immunization, the no immunization history (NH) group. The research number segment for the IH group was 001–326, and the research number segment for the NH group was 327–652. Participants were assigned research numbers in chronological order. Both groups were administered a two-dose immunization schedule of quadrivalent influenza vaccine on days 0 and 30. The inoculation site was the lateral deltoid of the upper arm, with the first dose being administered on the left arm and the second dose being administered on the right arm. All vaccines were of the same batch number but without additional numbering. The traceable code for the vaccine was used as the vaccine identity (ID) number. An Electronic Data Capture (EDC) system was used to capture and transmit data.

### 2.6. Evaluation of Immunogenicity

Prior to administering the first dose of the vaccine and 30 days after the first and second doses, 3 mL of venous blood was taken from each of the participants. A hemagglutination inhibition (HI) test was then used to detect the influenza virus HI antibody titer in the isolated serum. Immunogenicity was then evaluated based on the evaluation index as follows. (1) A HI antibody titer ≥1:40 was regarded as positive. (2) Seroconversion was defined as a HI antibody titer <10 on day 0 and a post-immunization HI antibody titer ≥40, or a HI antibody titer ≥10 on day 0 and a post-immunization HI antibody titer ≥4-fold increase in pre-immunization HI antibody titer. Then, we calculated and compared with the HI antibody positive rate, seroconversion rate, geometric mean titer (GMT), and the geometric mean fold-increase (GMI, GMI = post-immunization GMT/pre-immunization GMT). When the HI antibody titer was <1:10, a ratio of 1:5 was used to calculate the GMT.

This research was designed based on the guidelines of the National Medical Products Administration, Center for Drug Evaluation: Technical Guidelines for Clinical Studies of Seasonal Influenza Virus Vaccine (Draft for comment). In these guidelines, evaluate immunogenicity in individuals aged 3–60 years are (1) a seroconversion rate both sides of the 95% confidence interval (CI) lower limit ≥40%, (2) a positive rate (titer ≥ 1:40) both sides of the 95% CI lower limit ≥70% [6], and (3) a GMI both sides of the 95% CI lower limit ≥2.5 times, and these criteria were deemed acceptable for evaluating immunogenicity in our study. If the HI antibody of the H1N1, H3N2, BV, and BY strains of influenza virus in the IH and NH groups met the above criteria 30 days after the first-dose vaccination, it indicated that the immunogenicity of the one-dose full immunization schedule with the quadrivalent influenza vaccine was acceptable in children aged 3–8 years. If research data do not meet these criteria, the immunogenicity 30 days after the second dose should be evaluated.

### 2.7. Evaluation of Safety

To evaluate the safety of the vaccination schedule, participants were observed for the adverse event for 30 min after each dose of vaccination. Local (injection site) and systemic (non-injection site) adverse events were recorded and collected by using diary cards for 30 days after each vaccination. The local adverse events included pain, induration, swelling, rash, redness, itching, and cellulitis. The systemic adverse events included fever, diarrhea, constipation, dysphagia, anorexia, vomiting, nausea, arthralgia, headache, cough, dyspnea, skin and mucous membrane abnormality, provocation/inhibition, acute allergic reaction, fatigue, and other adverse events occurring during the clinical trial. Serious adverse events were collected by a combination of telephone follow-up once a month and spontaneous reporting from participants on days 31–180 after full vaccination.

According to the National Medical Products Administration No. 102 annunciation (2019 promulgation and implementation) Standard Guidelines for Grading Adverse Events in Clinical Trials of Prophylactic Vaccines [7], the grading of adverse events/reactions after vaccination is based on the degree of seriousness. Judgment of the correlation between an adverse event and vaccination was made according to the Five-level Classification Evaluation Standards of the National Center for ADR Monitoring. Five-level Classification Evaluation refers to the degree of causality between adverse events and vaccination and is defined according to the terms ‘certainly, probably, possibly, unlikely, and not related’. Adverse events related to vaccines are referred to as adverse reactions.

### 2.8. Statistical Analysis

The software SAS 9.4 was used for statistical analysis, with the significance level ***α*** set at 0.05. The confidence interval (CI) for rates was determined using the Clopper–Pearson exact probability method, whereas the CI for rate differences was determined using the normal approximation method.

Immunogenicity analysis: We calculated the seroconversion rate, positive rate (titer ≥ 1:40), GMI, and corresponding 95% CI of the strains H1N1, H3N2, BV, and BY of influenza HI antibody in the IH/NH groups 30 days after each dose of vaccine. We used the Clopper–Pearson exact probability method to calculate the 95%CI rate. We adopt a paired samples *t*-test to compare the differences in the GMT between 30 days after the first dose and 30 days after the second dose. For the analysis of differences in seroconversion and positive rates between 30 days after the first dose and 30 days after the second dose, we used McNemar’s test to compare.

Safety analysis: The Chi-square test, the corrected Chi-square test, or Fisher’s exact test were used for safety analysis to compare the incidence of adverse reactions and the distribution differences of the severity of adverse events/reactions (based on the number of cases) between the IH and NH groups.

Full analysis set of immunogenicity (FAS): All participants that conformed to the inclusion criteria, did not conform to the exclusion criteria, were included in the randomization, received at least one dose of vaccine, and had antibody tests before or after vaccination, were included in the FAS set. Immunogenicity per protocol set (PPS): All participants who conformed to the inclusion criteria, did not conform to the exclusion criteria, completed the entire vaccination program, had antibody tests before or after vaccination, and whose serum results were not eliminated by blind verification, were included in the PPS set. Safety set (SS): Participants who received vaccination after randomization were analyzed and evaluated for safety, and for whom any data violated the research protocol were not deleted, were included in the SS set. Those who completed the first-dose vaccination and safety observation were included in SST and SS1, and those who completed the second-dose vaccination and safety observation were included in SS2.

### 2.9. Serological Methods

Blood samples (about 3 mL) were collected on Days 0 (before vaccination), 30, and 60 from all available subjects. We used the hemagglutination inhibition (HI) assay to detect HI antibody titers at the National Institutes for Food and Drug Control. The HI assay is a standard and widely used measure to test HI titers, which is based on anti-hemagglutinin antibodies inhibiting the binding of the hemagglutinin of influenza viruses and red blood cells. 1% rooster red blood cells were incubated with serially diluted serum and standard influenza virus solution for 30 min. The antibody titer of hemagglutination inhibition is the highest dilution corresponding to hemagglutination inhibition completely.

## 3. Results

### 3.1. Basic Information on the Research Subjects 

A total of 652 recipients are enrolled in this research. The IH (immunization history) group comprised 329 participants, and the NH (non-immunization history) group comprised 323 participants. There were 166 males (50.46%) and 163 females (49.54%) in the IH group and 172 males (53.25%) and 151 females (46.75%) in the NH group. There was no statistical difference in the intergroup composition of the different sexes (*χ*^2^ = 0.510, *p* = 0.475). The average age in the two groups (average ± standard deviation) was 6.0 ± 1.7 years for the IH group and 5.1 ± 1.3 years for the NH group, which showed a statistical difference (t = 6.732, *p* < 0.001). The mean of these data was higher for the IH group than for the NH group, likely because of the requirement to receive two or more influenza vaccines in the IH group. However, these differences may result in a lower overall maturation of the immune system in the NH group than in the IH group.

**Figure 1:**
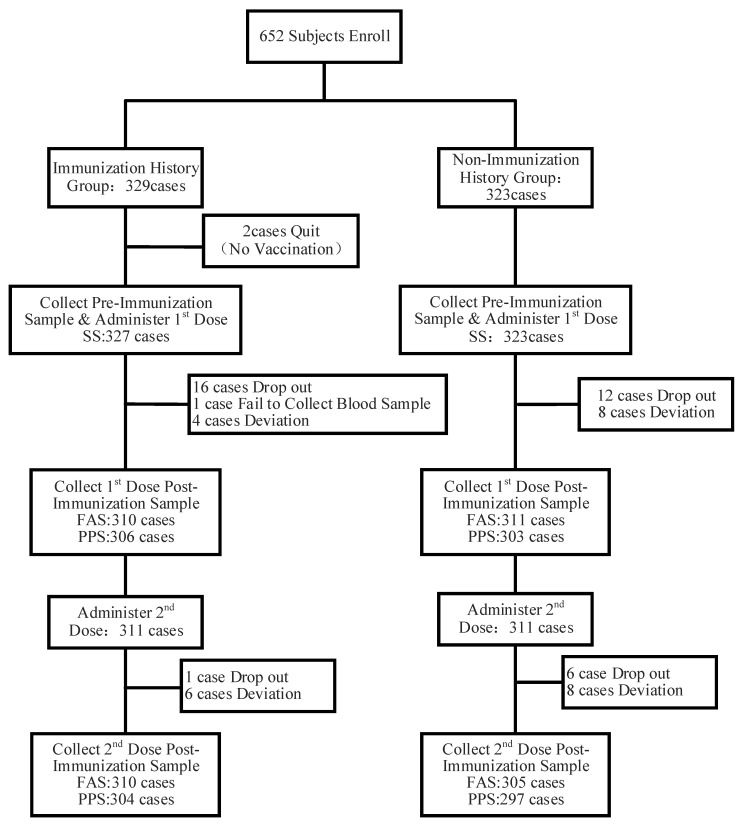
Participants disposition and study flow. SS, safety set; FAS, full analysis set; PPS, per protocol set.

There were 650 participants in both the SS set and the FAS set, of which there were 327 participants in the IH group and 323 participants in the NH group. There were 601 participants in the PPS set, of which there were 304 participants in the IH group and 297 participants in the NH group (Figure 1).

### 3.2. Immunogenicity

**Figure 2:**
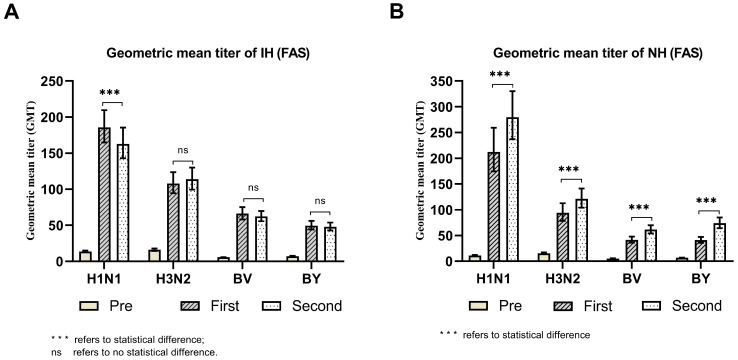
Comparisons of the GMT values of strains pre- and post-immunization with the first and second doses (FAS). Panel (**A**) Comparisons of GMT values of stains pre- and post-immunization with the first and second doses in the IH group (FAS). Panel (**B**) Comparisons of GMT values of strains pre- and post-immunization with the first and second doses in the NH group (FAS).

The baseline antibodies before immunization are shown in Table 1, Figure 2. The IH/NH group H1N1 strain antibody GMTs were 13.66 and 10.94, respectively, which showed an intergroup statistical difference (t = 2.957, *p* = 0.003); the H3N2 strain antibody GMTs were 16.11 and 15.36, respectively; the BV strain antibody GMTs were 5.68 and 5.47, respectively; and the BY strain antibody GMTs were 7.25 and 6.71, respectively. There was no statistical difference between the two groups in the pre-immunization GMTs of the H3N2, BV, and BY strains (*p* > 0.05).

A comparison of the GMTs of post-immunization antibodies is shown in Table 2, Figure 2. In the IH group, the GMTs of H1N1, H3N2, BV, and BY strain antibodies after the first-dose immunization were 185.95, 108.05, 66.26, and 49.72, respectively. Whereas the GMT antibodies to strains H1N1, H3N2, BV, and BY after the second-dose immunization were 162.90, 113.77, 62.37, and 48.08, respectively. When comparing the first-dose titers with the second-dose titers, only the difference in H1N1 antibody showed a statistically significant difference (t = 2.397, *p* = 0.017), and the post-immunization titer of the second dose was lower than that of the first dose. The differences in the antibodies to strains H3N2, BV, and BY were not statistically significant (*p* > 0.05).

In the NH group, the GMTs of H1N1, H3N2, BV, and BY strain antibodies after the first-dose immunization were 212.57, 94.01, 41.48, and 40.92, respectively. Whereas the GMT antibodies to strains H1N1, H3N2, BV, and BY after the second-dose immunization were 279.85, 121.26, 61.46, and 73.88, respectively. When comparing the first-dose titers with the second-dose titers, GMT antibodies to strains H1N1, H3N2, BV, and BY showed statistical differences (all *p* ≤ 0.001), confirming that the post-immunization titers of the second dose were higher than those of the first dose.

**Figure 3:**
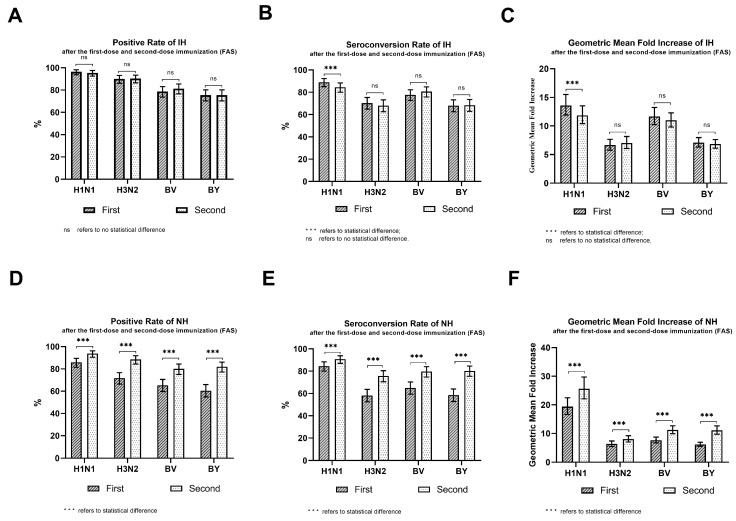
Comparison of the immunogenicity of each strain 30 days after the first-dose and second-dose immunizations. Panel (**A**) Positive Rate after the first-dose and the second-dose immunization in the IH group (FAS). Panel (**B**) Seroconversion Rate after the first-dose and the second-dose immunization in the IH group (FAS). Panel (**C**) Geometric Mean Fold Increase after the first-dose and the second-dose immunization in the IH group (FAS). Panel (**D**) Positive Rate after the first-dose and the second-dose immunization in the NH group (FAS). Panel (**E**) Seroconversion Rate after the first-dose and the second-dose immunization in the NH group (FAS). Panel (**F**) Geometric Mean Fold Increase after the first-dose and the second-dose immunization in the NH group (FAS).

The post-vaccination antibody positive rates, the seroconversion rates, and the GMI values are shown in Table 3, Figure 3. In the IH group, after the first-dose immunization, the positive rates for H1N1, H3N2, BV, and BY strain antibodies were 96.45%, 90.00%, 78.71%, and 75.48%, respectively, and the lower limits of the two-sided 95% CI were ≥70%. The seroconversion rates (SCR) for the antibodies of strains H1N1, H3N2, BV, and BY were 89.03%, 70.32%, 77.74%, and 68.06%, respectively, and the lower limits of the two-sided 95% CI were ≥40%. The GMI values of the antibodies of strains H1N1, H3N2, BV, and BY were 13.59, 6.66, 11.65, and 7.09 times, respectively, and the lower limits of the two-sided 95% CI were ≥2.5 times. The positive rates, seroconversion rates, and GMI values for antibodies of strains H1N1, H3N2, BV, and BY after the second-dose immunization were similar to those after the first-dose immunization. In the IH group, the positive rates, seroconversion rates, and GMI values after both the first and second-dose immunizations all met the acceptable criteria for immunogenicity [6] for strains H1N1, H3N2, BV, and BY. There were no statistical differences in the positive rates, seroconversion rates, or GMI values post-immunization after the first dose and second dose (*p* > 0.05) for strains H3N2, BV, and BY. Only the seroconversion rate and the GMI value for the H1N1 strain showed a statistically significant difference (*p* < 0.05), with the values being lower after the second dose than after the first dose of immunization.

In the NH group, after the first-dose immunization, the positive rates for the H1N1, H3N2, BV, and BY strain antibodies were 85.85%, 71.70%, 65.27%, and 60.45%, respectively. The 95% CI lower limit of the H3N2, BV, and BY strain positive rates was <70%, which failed to meet the acceptable criteria of immunogenicity [6]. However, 30 days after the second-dose vaccination, the positive rates of antibodies to the strains H1N1, H3N2, BV, and BY were 93.77%, 88.52%, 80.00%, and 81.97%, respectively, and the lower limits of the two-sided 95% CI were ≥70%. The seroconversion rates were 90.82%, 75.74%, 79.67%, and 80.33%, respectively, and the lower limits of the two-sided 95% CI were ≥40%. The GMI values were 25.61, 8.02, 11.20, and 11.07 times, respectively, and the lower limits of the two-sided 95% CI were ≥2.5 times. In the NH group, the positive rates, seroconversion rates, and GMI values after the second-dose immunization all met the acceptable criteria for immunogenicity [6]. There were statistical differences in the positive rates, seroconversion rates, and GMI values post-immunization between the first and second doses for strains H1N1, H3N2, BV, and BY (*p* < 0.05), and the values were higher after the second dose than after the first dose.

### 3.3. Safety Analysis

As shown in Table 4, from the first-dose vaccination to 30 days after the complete vaccination program, the incidence rates of adverse reactions in the IH and NH groups were 20.80% and 19.50%, respectively, for which the intergroup difference was not statistically significant (*p* > 0.05). The incidence rates of local adverse reactions in the two groups were 13.46% and 8.98%, which also did not show a statistically significant intergroup difference (*p* > 0.05). The incidence rates of systemic adverse reactions in the two groups were 8.56% and 11.76%, with no statistically significant intergroup difference (*p* > 0.05).

As shown in Table 5, based on the classification of the severity of total adverse reactions in the two groups, the differences between the groups were not statistically significant (*p* > 0.05). Based on the classification of severity of local adverse reactions in the two groups, the differences between the groups were statistically significant (*p* < 0.05). There was no significant difference in the classification of severity of systemic adverse reactions between the two groups (*p* > 0.05).

Since the IH and NH groups showed similar safety characteristics, we merged data on the adverse reactions of the two groups. According to the identification criteria of incidence recommended by the Council for International Organizations of Medical Sciences (CIOMS), the symptoms of adverse reactions in all recipients that occurred during the full course of vaccination were counted. Common symptoms (1–10%, including 1%) included injection site swelling (6.31%), injection site pain (6.15%), cough (4.46%), injection site spot-erythema (4.15%), fever (3.85%), and injection site itching (1.08%). Occasional symptoms (0.1–1.0%, including 0.1%) included runny nose (0.62%), diarrhea (0.62%), headache (0.46%), arthralgia (0.31%), fatigue (0.31%), vomiting (0.31%), parotid enlargement (0.31%), salivary gland pain (0.31%), loss of appetite (0.15%), myalgia (0.15%), epistaxis (0.15%), oropharyngeal pain (0.15%), erythema (0.15%), erythematous eruption (0.15%), itching (0.15%), injection site rash (0.15%), constipation (0.15%), and nausea (0.15%).

From the first-dose vaccination to 31–180 days after the complete vaccination program in the IH and NH groups, no serious adverse events related to vaccination were reported.

## 4. Discussion

Influenza is an infectious disease requiring global surveillance [8]. Vaccination is recognized as the most effective method to prevent influenza. Adopting an immunization schedule based on the most recent scientific research for specific groups of individuals will help to ensure total protection. It has been shown that vaccinating children against influenza virus can increase their resistance [9]. However, the optimal vaccination regimen has not yet been precisely defined.

We found that the average age in our study showed a statistical difference (t = 6.732, *p* < 0.001) in the IH and NH groups. The mean of these data was higher for the IH group than the NH group, likely because of the requirement to receive two or more influenza vaccines in the IH group. The main purpose of this study was to determine the optimal vaccination program for children aged 3–8 years by comparing the differences in immune response post-immunization following one or two doses of vaccine in the IH and NH groups, respectively, rather than comparing the difference in immune response between the two groups. Therefore, even if the difference in the average age of the two groups were significant, it would not affect the scientific validity or accuracy of the final conclusions of this study.

Our findings revealed that before vaccination, the GMTs of H1N1 in the IH group were slightly higher than those in the NH group, with no significant differences in H3N2, BV, and BY. This phenomenon may indicate that some antigens remain from previous influenza vaccination in the IH group because they have a high HI antibody titer of the H1N1 strain influenza virus after inoculation with influenza vaccine containing the H1N1 strain antigen. This is consistent with the findings of Chen et al. The immunogenicity of IIV4 in 3–8 year old individuals may be influenced by their pre-immune antibody titers [10]. After the first dose, the positive rate, seroconversion rate, and GMI value for the H1N1, H3N2, BV, and BY antibodies in the IH group all met the acceptable criteria of immunogenicity [6]. After the second dose, there was a statistically significant difference in the GMT of H1N1 between the first and second post-immunization levels (t = 2.397, *p* = 0.017), and the GMT after the second-dose vaccination was lower than after the first-dose vaccination. The difference in the GMT after the first and second-dose immunization for the strains H3N2, BV, and BY was not statistically significant (*p* > 0.05). This may be due to the fact that people with a history of immunization have “residual” antibodies and immune memory prior to the first vaccination. Thirty days after the first vaccination, the antibodies to each strain have already peaked, and a second vaccination within a short period of time (30-day interval) will not be able to produce a higher level of antibodies or even cause the high level of H1N1 antibodies to decrease. This means that, in this population, immunization with one dose of quadrivalent influenza vaccine will induce sufficient antibody levels, and a second dose is not needed.

Fu et al. conducted a survey of children aged 6 months to 8 years who received the influenza vaccine and found that the efficacy of one dose, administered during the influenza season, was too low and further booster doses were needed [11]. This was consistent with the results of the present study, in which the group with no history of immunization had a rate of positivity less than optimal for BV and BY antibodies after one dose of vaccine. After the second dose, the GMT values of the strains H1N1, H3N2, BV, and BY increased significantly, and the positive rate and seroconversion rate both reached the acceptable criteria of immunogenicity [6], indicating more comprehensive protection. These findings suggest that children aged 3–8 years with no immunization history should be administered two doses of quadrivalent influenza vaccine ≥4 weeks apart to achieve the best immunization effect. This conclusion is consistent with a retrospective cohort study to evaluate the protective effect of the influenza vaccine carried out by Ritzwoller et al. [12]. A study by Chen et al. also reaffirmed the idea that two doses are more effective than one [10]. In addition, Neuzil et al. conducted research to evaluate the necessity of two doses of influenza vaccine in older children aged 5–8. Their results also showed that administering two doses of influenza vaccine can increase the antibody titer of strains H1N1, H3N2, BV, and BY to a high level for children without an influenza vaccine immunization history. The two-dose schedule is, therefore, considered the best strategy to prevent influenza [13]. Zhang et al. administered a second booster dose to children aged 3–8 years on day 28 after the first dose of vaccine and found that the booster provided better seroprotection to the children compared with only one dose of vaccine [14].

Safety is an important indicator of vaccine production. Thime et al. found that no serious unsolicited adverse events or vaccine-related SAEs were reported in the Phase III clinical trial [15]. When the vaccine was administered to children, the most common reaction was pain and tenderness at the injection site, but this subsided within 3 days, and there was only one unsolicited adverse reaction associated with the vaccine, but these were Grade 2 or 4 transient [9]. In the present study, individuals in the NH group were vaccinated according to a two-dose schedule. The total adverse reaction incidence was 19.50%. The incidence of adverse reactions after the first-dose vaccination was 13.31%, compared with 8.68% after the second-dose vaccination. The adverse reactions were all Grade 1 or Grade 2. No Grade 3 or higher adverse reactions were detected. The symptoms with the highest incidence rate were pain at the injection site (2.25%) and fever (1.29%). All of these data show that the quadrivalent influenza vaccine produced by Hualan Bio is acceptable and has favorable safety for 3–8-year-old children without an immunization history to be vaccinated with two doses ≥4 weeks apart.

The symptoms and incidence rates of adverse reactions in all recipients that occurred in this study are consistent with the results of previous studies in which the common adverse reaction found was pressure or pain at the injection site [9]. Influenza is an acute respiratory infectious disease. Compared with adults, children have a higher incidence of influenza and show a longer infection time, which tends to promote the spread of the influenza virus, especially in schools and among families. To reduce the risk of influenza in children and their families, it is suggested that the population be classified carefully with regard to the vaccination strategy. This is the first study of this type to be conducted in China, and the conclusions of this study are consistent with the immunization schedule of influenza vaccine in children aged 3–8 years recommended by the ‘Prevention and Control of Seasonal Influenza with Vaccines: Recommendations of the Advisory Committee on Immunization Practices [5], United States’ guidelines published by the Center for Disease Control and Prevention. For children aged 3–8 years with no influenza immunization history, an immunization schedule of two doses ≥4 weeks apart is recommended to gain optimal protection. The results of this study, which provide a better immunization schedule, will result in better protection and reduce medical and economic losses for world health security.

## 5. Conclusions

In summary, this study showed that the quadrivalent influenza split-virion vaccine is highly immunogenic and generally safe in children aged 3–8 years. An influenza immunization schedule of two doses of quadrivalent influenza vaccine ≥4 weeks apart is recommended to gain optimal protection for children aged 3–8 years with no influenza immunization history.

## Figures and Tables

**Table 1:** Pre-immunization antibody GMTs comparisons between the IH group and NH group (FAS).

Antibody Strain	Group	N	Min, Max	M(P_25_, P_75_)	GMT(95% CI)	t	*p*
H1N1	IH	327	5, 160	10(5, 40)	13.66(12.30~15.16)	2.957	0.003
	NH	323	5, 160	5(5, 20)	10.94(9.87~12.14)		
H3N2	IH	327	5, 320	20(5, 40)	16.11(14.51~17.89)	0.644	0.520
	NH	323	5, 320	20(5, 40)	15.36(13.88~17.00)		
BV	IH	327	5, 40	5(5, 5)	5.68(5.47~5.89)	1.470	0.142
	NH	323	5, 40	5(5, 5)	5.47(5.29~5.65)		
BY	IH	327	5, 80	5(5, 10)	7.25(6.76~7.77)	1.592	0.112
	NH	323	5, 160	5(5, 5)	6.71(6.29~7.16)		

N, Number. Min, Minimum antibody titer. Max, Maximum antibody titer. GMT, the geometric mean titer.

**Table 2:** Comparison of the antibody GMTs 30 days after the first-dose and second-dose immunization (FAS).

Group	Antibody Strain	Dose	N *	Min, Max	M(P_25_, P_75_)	GMT(95% CI)	t	*p*
IH	H1N1	1st dose	309	10, 2560	160(80, 320)	185.95(164.82~209.79)	2.397	0.017
		2nd dose	309	10, 2560	160(80, 320)	162.90(142.96~185.61)		
	H3N2	1st dose	309	10, 5120	80(40, 320)	108.05(94.40~123.68)	0.793	0.429
		2nd dose	309	10, 5120	80(40, 160)	113.77(99.53~130.06)		
	BV	1st dose	309	5, 1280	80(40, 160)	66.26(58.27~75.35)	1.079	0.282
		2nd dose	309	5, 640	80(40, 160)	62.37(55.70~69.83)		
	BY	1st dose	309	5, 1280	40(40, 80)	49.72(43.99~56.21)	0.688	0.492
		2nd dose	309	5, 1280	40(40, 80)	48.08(42.97~53.80)		
NH	H1N1	1st dose	305	5, 5120	320(40, 1280)	212.57(174.44~259.03)	3.357	0.001
		2nd dose	305	5, 5120	320(80, 1280)	279.85(237.12~330.26)		
	H3N2	1st dose	305	5, 5120	80(20, 320)	94.01(78.32~112.85)	3.293	0.001
		2nd dose	305	5, 5120	160(40, 320)	121.26(104.08~141.27)		
	BV	1st dose	305	5, 1280	40(20, 80)	41.48(35.94~47.88)	6.760	<0.001
		2nd dose	305	5, 1280	80(40, 160)	61.46(54.01~69.94)		
	BY	1st dose	305	5, 1280	40(20, 80)	40.92(35.60~47.03)	8.215	<0.001
		2nd dose	305	5, 1280	80(40, 160)	73.88(64.30~84.89)		

* Because Table 2 shows a statistical comparison between the first and the second vaccinations, “N” in the table refers to the number of participants from whom blood samples were collected and serum results were obtained 30 days after the first-dose and second-dose vaccinations.

**Table 3:** Immunogenicity for each strain 30 days after the first-dose and second-dose immunizations (FAS).

Group	Strain	Dose	N	Positive Number	Positive Rate	Seroconversion Number	Seroconversion Rate	GMI *
IH	H1N1	1st	310	299	96.45(93.74~98.22)	276	89.03(85.01~92.28)	13.59(11.91~15.50)
		2nd	310	296	95.48(92.54~97.51)	262	84.52(80.00~88.36)	11.86(10.40~13.53)
	H3N2	1st	310	279	90.00(86.11~93.10)	218	70.32(64.90~75.35)	6.66(5.78~7.68)
		2nd	310	280	90.32(86.47~93.38)	211	68.06(62.56~73.22)	7.03(6.05~8.16)
	BV	1st	310	244	78.71(73.73~83.13)	241	77.74(72.70~82.25)	11.65(10.24~13.24)
		2nd	310	252	81.29(76.50~85.48)	250	80.65(75.80~84.89)	10.99(9.82~12.29)
	BY	1st	310	234	75.48(70.30~80.17)	211	68.06(62.56~73.22)	7.09(6.31~7.97)
		2nd	310	234	75.48(70.30~80.17)	212	68.39(62.89~73.53)	6.84(6.13~7.63)
NH	H1N1	1st	311	267	85.85(81.48~89.53)	263	84.57(80.06~88.40)	19.34(16.65~22.46)
		2nd	305	286	93.77(90.44~96.21)	277	90.82(87.01~93.81)	25.61(22.07~29.73)
	H3N2	1st	311	223	71.70(66.35~76.64)	181	58.20(52.50~63.74)	6.30(5.41~7.34)
		2nd	305	270	88.52(84.40~91.88)	231	75.74(70.53~80.44)	8.02(6.98~9.21)
	BV	1st	311	203	65.27(59.70~70.56)	202	64.95(59.37~70.25)	7.62(6.67~8.70)
		2nd	305	244	80.00(75.06~84.34)	243	79.67(74.71~84.04)	11.20(9.89~12.68)
	BY	1st	311	188	60.45(54.78~65.92)	182	58.52(52.82~64.05)	6.16(5.49~6.92)
		2nd	305	250	81.97(77.18~86.12)	245	80.33(75.42~84.64)	11.07(9.70~12.64)

* Geometric mean fold-increase, GMI = post-immunization GMT/pre-immunization GMT.

**Table 4:** Comparison of the incidence of overall adverse reactions after vaccination between the IH and NH groups (SS).

AEs	Group	N	* Times	# Cases	Rate (95% CI)	X^2^	*p*
Total Adverse Reaction	IH	327	111	68	20.80(16.53~25.60)	0.168	0.682
	NH	323	102	63	19.50(15.33~24.25)		
	Total	650	213	131	20.15(17.13~23.45)		
Local Adverse Reaction	IH	327	74	44	13.46(9.95~17.64)	3.267	0.071
	NH	323	49	29	8.98(6.10~12.64)		
	Total	650	123	73	11.23(8.91~13.91)		
Systemic Adverse Reaction	IH	327	37	28	8.56(5.77~12.14)	1.826	0.177
	NH	323	53	38	11.76(8.46~15.79)		
	Total	650	90	66	10.15(7.94~12.74)		

* The number of symptoms of adverse reactions occurring after each vaccination; # The number of subjects with adverse reactions after vaccination.

**Table 5:** Composition of the degree of adverse reactions in the IH and NH groups (based on frequency).

AEs	Group	Times	Grade 1	Grade 2	Grade 3	X^2^	*p*
Total Adverse Reaction	IH	111	75	34	2		0.518 ^a^
	NH	102	74	28	0		
	Total	213	149	62	2		
Local Adverse Reaction	IH	74	58	15	1		0.003 ^a^
	NH	49	48	1	0		
	Total	123	106	16	1		
Systemic Adverse Reaction	IH	37	17	19	1		0.644 ^a^
	NH	53	26	27	0		
	Total	90	43	46	1		

^a^: Fisher’s exact test.

## Data Availability

The data presented in this report are available on request from the corresponding author. The data are not publicly available due to restrictions concerning privacy or ethical considerations.
